# Pathways into and out of overweight and obesity from infancy to mid‐childhood

**DOI:** 10.1111/ijpo.12427

**Published:** 2018-07-11

**Authors:** C. M. Wright, L. Marryat, J. McColl, U. Harjunmaa, T. J. Cole

**Affiliations:** ^1^ School of Medicine, College of MVLS University of Glasgow Glasgow UK; ^2^ School of Mathematics and Statistics University of Glasgow Glasgow UK; ^3^ Farr Institute @ Scotland/Scottish Collaboration for Public Health Research and Policy University of Edinburgh Edinburgh UK; ^4^ Center for Child Health Research University of Tampere Faculty of Medicine and Life Sciences, and Tampere University Hospital Tampere Finland; ^5^ Population, Policy and Practice Programme UCL Great Ormond Street Institute of Child Health London UK

**Keywords:** Infancy, obesity, overweight, tracking

## Abstract

**Objectives:**

To investigate whether high weight in infancy predicts obesity in childhood.

**Method:**

Data from two UK cohorts (Newcastle Growth and Development *N* = 795, Gateshead Millennium *N* = 393) and one Finnish (Tampere *N* = 1262) were combined. Z scores of weight at 3 and 12 months and body mass index (BMI) at 5 and 8 years were categorized as raised/overweight (1 to <2 SD) or high/obese (≥2 SD).

**Results:**

The majority of infants with raised or high weight at birth tended to revert to normal by 3 months and to track in the same category from 3 to 12 months. Although infants with high weight were five times more likely to have BMI ≥ 2 SD at 8 years (*p* < 0.001), only 22% went on to have BMI ≥ 2 SD, while 64% of infants with raised weight had normal BMI at 8 years. Of children with BMI ≥ 2 SD aged 8 years, only 22% had raised weight in infancy and half had BMI ≥ 2 SD for the first time at that age.

**Conclusions:**

Infants with raised weight in infancy tend to remain so, but most children who go on to have BMI ≥ 2 SD were not unusually heavy infants and the majority of infants with high weight reverted to overweight or normal weight in childhood.

## Introduction

While increasing levels of overweight and obesity throughout the life course are concerning, worries about overweight and obesity in childhood cause particular anxiety, due to concern that this will fuel even larger rises in rates of obesity in later life 1. Many studies have described an association between overweight and rapid weight gain in infancy with overweight in later childhood 2, 3, 4, 5. This has led to the proposal that obesity has its origins in early childhood and that infancy, or even pregnancy, could be a prime period in which to prevent obesity, as it precedes the onset of obesity for most children and the child's diet is largely under parental control 6, 7. However, evidence from early life preventative interventions has generally been disappointing 8 and some have suggested that this approach is not realistic, both because of the difficulty of actually changing early risk factors for obesity and the relative lack of tracking from infancy to childhood 9. This apparent paradox reflects the fact that, while there undoubtedly is an association between infant weight and later overweight, it is not clear how important that association really is. Thus, a German study found that while weight change over the first 2 years of life was the best predictor of a body mass index (BMI) in the obese range at school entry, still only one in five children with rapid weight gain went on to have obesity 4 and we made a similar observation in a UK cohort 10.

However, our ability to track the growth of individuals over time is limited by the availability of good quality datasets with regular measurements. Most cohorts, particularly historic ones, have included relatively few children with BMI well above the healthy range. It may be that children with extreme weight at early ages are on a trajectory towards later obesity, but most cohorts would not be large enough to detect this.

We thus aimed to bring together data from three comparable cohorts, including the cohort cited earlier, to form a dataset large enough to allow us to properly investigate the pathways that children take into and out of overweight and obesity between birth and 8 years. In particular, the paper aims to clarify the extent to which clinicians should be concerned about individual infants with high weights.

## Methods

### Datasets

Data came from existing, population‐representative, longitudinal growth studies, retrieved mainly from routine records. They had already been cleaned, checked and analysed for other purposes, with all studies already published. Details of the studies are as follows.

### Newcastle Growth and Development Study (1987)

This dataset comprises the routine weights of a birth cohort of 3418 children born at term in Newcastle upon Tyne between June 1987 and May 1988. Up to 11 weights measured by clinical staff in infancy were retrieved from baby clinic records and 3060 of the babies had at least two weights. No heights were recorded at this stage 11. At school age, a 20% systematic sample of the 2812 (82%) children for whom at least three weights had been retrieved was drawn, of whom 448 children (80%) were traced and had height and weight measured at 8–9 years 12. In addition, heights and weights for this sample were retrieved from school entry growth check data at age 4–5, where possible.

### Gateshead Millennium Study (1999)

Gateshead Millennium Study is a birth cohort of 1029 babies (923 term) born in Gateshead in 1999–2000, representing 81% of eligible births during the recruitment period. Routine weights were retrieved from baby clinic records. There was a mean of 13 weights per child in the first year, and length was collected by research nurses for 830 infants at age 13 months. Routine weights measured at school entry (age 4–5) were retrieved from routine data records (mean age 4.9). Data were further collected from 585 children at age 6–8 years at school and/or home visits (mean age 7.5) 10, 13.

### Tampere study, Finland (2003)

This dataset comprises the routine heights and weights of 2809 children aged 0–4 years born between October 2003 and September 2004 who attended child health clinics in Tampere. Children were weighed and measured by clinical staff on electronic scales. Up to 16 scheduled events were recorded per child from birth to 5 years. There was a mean of 12 weight and length/height measurements per child 14. Further data were collected on 1935 children at follow‐up between 6 and 8.4 years (mean 7.2).

### Analysis

Weights were converted to age and sex‐adjusted Z scores. BMI was not used in infancy, mainly due to a lack of universal length data but also due to the lack of validity in using BMI at this young age. The WHO 2006 standard was used for the infancy Z scores 15, while the UK 1990 reference was used at age 4–5 and 7–9 16. The latter reference was used for older children as the WHO standard stops at 5 years and the WHO reference, for 5–18 years, is based on US data, where children are heavier than in the UK.

For each child, the measurements nearest to the target ages of 3, 6, 9 and 12 months within an age band of ±1.5 months were identified. Similarly, measurements for each child nearest to the target ages of 5 and 8 years were selected within the age bands of 4.0 to 6.0 and 6.5 to 9.0 years, respectively.

We used categorical rather than continuous outcomes because the BMI distribution is non‐normal and variation within the normal range is less predictive of variation in adiposity than variation in the upper tail of the distribution 17. We selected widely used cut‐offs that correspond to the Z score distribution as follows:

1. Raised weight/overweight BMI: Z score between 1 and <2, which equates to the 85th centile on the UK 1990 reference and corresponds roughly to the International Obesity Task Force overweight threshold, which itself is extrapolated from the adult overweight threshold of BMI 25 kg m^−2^
2. High weight/obese BMI: Z score two or above, equating to the 97/98th centile on the UK 1990 reference, which is slightly lower than the International Obesity Task Force obesity threshold.

The first phase of work explored the stability of weight during the first year of life. Levels of raised and high weight at each time point were described. Models were fitted to investigate the likelihood of moving between weight states (i.e. normal weight, overweight and obesity) over 3‐monthly periods during the first year using the statistical programming language R 18. The models calculate the conditional probability of children moving from one weight state to another, to quantify the likely pathways that children take through time. The probability of moving between weight states from one time point to the next can be presented as a probability transition matrix 19. Before merging the datasets, we tested whether their probability transition matrices differed significantly, using a generalized likelihood ratio test. They did not (data not shown), so the datasets were pooled.

Pathways to raised BMI in childhood were then explored. Using binary variables of raised or normal weight or BMI at each time point, we looked at movement between weight states from 3 months to 8 years.

## Results

At 12 months, 5% of infants had high weight (≥2 SD) and 26% raised or high weight (≥1 SD). At 5 years, 4% of children had BMI ≥ 2 SD and 21% had BMI ≥ 1 SD, while by 8 years, 6% had BMI ≥ 2 SD and 23% had BMI ≥ 1 SD. Rates of high weight at 12 months varied from 2% to 8% by cohort and gender, while 16–31% had raised or high weight. The rates of obesity at 5 and 8 years varied from 2% to 9%, while combined overweight and obesity varied from 14% to 35% at 5 years and 17–28% at 8 years (Table s1). There were around 2400 children with data at each age.

Infants with raised or high weight at birth showed a strong tendency to regress towards normal weight by 3 months (Table 1). For example, in girls, 74% of those with raised weight and 54% of those with high weight at birth had reverted to normal weight by 3 months. After 3 months, weight status was much more stable, so for example, only 13% and 1% of girls with raised and high weight respectively at 3 months had reverted to normal by 6 months.

**Table 1:** Transition matrices for girls and boys across weight categories in each status over 3‐month interval intervals by sex

	Start weight status	End weight status					
		Girls			Boys		
		Normal	Raised	High	Normal	Raised	High
0–3 m	Normal	0.94	0.05	0.01	0.92	0.08	0.01
	Raised	0.74	0.25	0.01	0.67	0.28	0.04
	High	0.54	0.36	0.09	0.49	0.38	0.13
3–6 m	Normal	0.86	0.13	0.01	0.86	0.12	0.01
	Raised	0.13	0.75	0.13	0.14	0.71	0.16
	High	0.01	0.10	0.89	0.01	0.09	0.90
6–9 m	Normal	0.91	0.08	0.01	0.89	0.11	0.01
	Raised	0.14	0.77	0.08	0.18	0.70	0.13
	High	0.02	0.21	0.77	0.02	0.16	0.82
9–12 m	Normal	0.91	0.09	<0.01	0.90	0.10	0.01
	Raised	0.19	0.74	0.07	0.19	0.73	0.09
	High	0.04	0.28	0.68	0.03	0.25	0.72

Values are probabilities, adding to 1 in each row. Each cell indicates the probability of a child starting in that row category and ending in that column category 3 months later.

Weights showed moderate correlations from age to age within infancy and BMI at age 5 and 8 years were strongly correlated, but there was only weak correlation between infancy weight and childhood BMI (Table S2).

### Pathways from raised or high weight in infancy

Due to the large amount of centile crossing in early infancy, 3 months rather than birth was used as the baseline to investigate infant–child transitions. Altogether, 29% (620/2122) of children with both infancy and childhood data had a raised or high weight at 3 and/or 12 months (Table 2). These children were significantly more likely to go on to have high BMI in childhood. Those with high weight ever in infancy (i.e. at 3 and/or 12 months) were 5.4 (95% CI 3.6, 8.3) times more likely to have BMI ≥ 2 SD and 3.77 (3.10, 4.58) times more likely to have BMI ≥ 1 SD than those who never had a high weight. Children with raised weight in infancy were equally likely to go on to have BMI ≥ 2 SD (relative risk 2.30 [1.59, 3.34]) as to have BMI ≥ 1 SD (relative risk 2.37 [2.01, 2.80]) compared with those who never had a raised or high weight.

**Table 2:** Overweight or obesity status for children at 8 years by weight status in infancy

Weight status in infancy	BMI status at 8 years % (*n*)			Total
	Normal	Overweight	Obese	
Never raised	85 (1273)	11 (169)	4 (60)	71 (1502)
Ever raised but never high	64 (319)	27 (135)	9 (46)	24 (500)
Ever high	42 (51)	36 (43)	22 (26)	6 (120)

However, nearly two‐thirds of infants with raised weight and nearly half of those with high weight ever in infancy did not have BMI ≥ 1 SD at 8 years, while fewer than a quarter of those ever high weight in infancy later had BMI ≥ 2 SD. The transition patterns were similar for boys and girls.

### Pathways to overweight and obesity at 8 years

Focussing on the 1605 children with measurements at all four target ages, 361 had BMI ≥ 1 SD at 8 years, of which 98 children had BMI ≥ 2 SD. Of these, 57% had raised weight in infancy at either or both 3 or 12 months and 19% had always had raised weight or BMI ≥ 1 SD from 3 months (Table 3). However, over 40% of the children who had BMI ≥ 1 SD at age 8 had never had raised or high weight in infancy. Of the children who had BMI ≥ 2 SD at age 8, 78% had not had high weight in infancy and 52% had not previously had a high weight or BMI ≥ 2 SD (Table 3). Only five (0.3%) of all children with BMI ≥ 2 SD at 8 years had consistently high weights and/BMI from infancy.

**Table 3:**
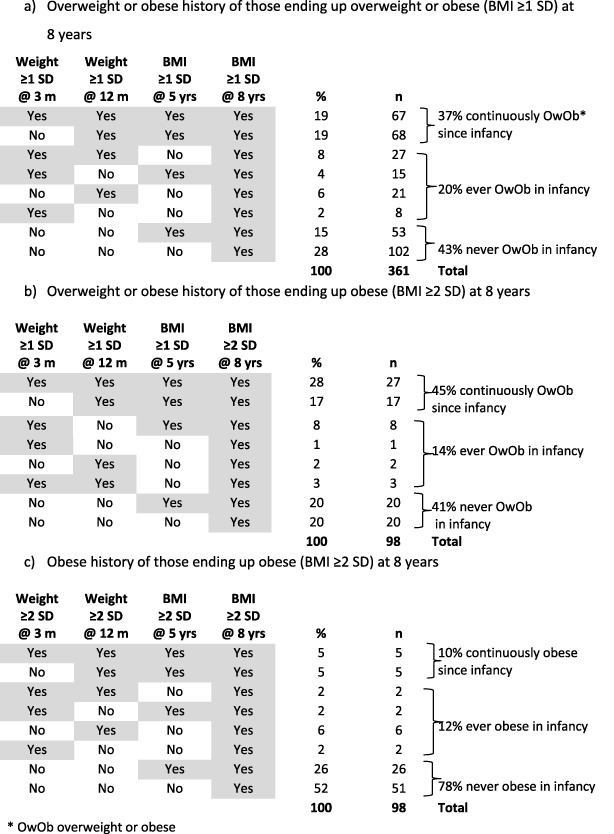
Previous history of overweight or obesity for children overweight or obese at 8 years

## Discussion

The novel feature of this study is the large number of children with weights both in infancy and mid‐childhood. Few previous studies have had sufficient numbers over a long enough period to allow assessment of the extent to which an extreme weight in infancy should excite anxiety about future obesity. Most studies have, as a result, used overweight or a relatively low ‘obesity’ threshold as both a risk factor and an outcome.

In this study, raised weight in infancy (SD ≥ 1) was a significant risk factor for later overweight, and once a child had become overweight, this tended to persist, with three quarters of those overweight at 8 years having been overweight earlier. This has been described previously. A large systematic review found odds ratios for later overweight varying from 1.35 to 9.38, reflecting the varied definitions of obesity used 2. A study by Pan et al. of a large US population cohort 20 found that those with weight‐for‐height above the 95th centile before age 2 years had double the risk of being above the 95th BMI centile 2–3 years later, while another large population‐based study by Mei et al. found that infants with weight‐for‐height above the 95th centile were three times more likely to be above the 95th centile 3 years later 21. For those infants above our more stringent threshold of high weight in infancy (≥2 SD or 98th centile), we found a fivefold increased risk of obesity 7 years later.

However, we also found that over half the infants who were overweight had normal BMI by 8 years. This is also consistent with Pan's study 20, where almost the same number of children reverted to a normal BMI over 2 years as became obese, and Mei's study where 37% reverted to non‐overweight after 3 years. In our study, where we had relatively large numbers of infants with high weight, three quarters had BMI < 2 SD by 8 years and the great majority of those with BMI ≥ 2 SD at 8 years had not had high weight in infancy. This suggests that, while overweight may be an enduring characteristic, more extreme BMIs in infancy tend not to persist. Thus, a high weight in infancy has a moderate positive predictive value for becoming overweight in mid‐childhood, but not for becoming obese, and its sensitivity is very low.

This study has the unusual advantage of combining three longitudinal growth cohorts, with regular measurements throughout infancy and follow‐up data in later childhood. The datasets used were all of high quality and representative of their populations. While two of the three studies were from the UK, the most recent study was from Finland, where growth patterns could differ, although rates of childhood obesity are fairly similar between the two countries 22. Although we had sufficient numbers to look at weight transitions during infancy, a limitation is that we did not have enough height data to look at BMI transitions in infancy. As a result, we cannot know how much the variation in weight reflected changes in adiposity and how much stature. In practice, this is less valid for younger children, where it is weight that is usually monitored, not BMI.

During the first 3 months, there was substantial regression to the mean for children born heavy. Because of this, we used 3 months rather than birth as a baseline. A previous study has noted that birthweight bears little relation to weight at 5 years 23, but another recent study found an association between high birthweight and raised BMI in mid‐childhood 24. However, it is not clear whether this association reflects increased fat mass, or just tracking of lean mass, because another recent study found that BMI at 9 months was associated only with fat free mass in adulthood 25. It has been argued 26, 27 that the period from birth to 2 years is one of the most critical in predicting later obesity, but most studies examining this issue have emphasized the significance of associations over time, rather than the effect sizes, which are small. While it is clear that there is a statistical association between high weight in infancy and later overweight, another group has also recently noted the limited tracking of overweight from 2 years to school entry, although that group lacked data from infancy and could only examine rates of obesity and overweight combined 28.

When one combines the evidence from this larger analysis with the observation that the variance of BMI in infancy seems to mainly reflect tracking of lean mass 25, 29, this suggests a need to accept that ‘obesity’ in infancy is not in fact an important risk factor for obesity in childhood. As children begin to interact more with the obesogenic environment, those with infancy weight above the normal range will be at some increased risk of later obesity, but most children with high BMI in childhood will emerge from the much larger group of infants with normal weight. Thus, a watch and wait approach seems most sensible in infancy, while continuing universal interventions, such as those promoting exclusive breastfeeding, supporting healthy complementary feeding and encouraging physical activity.

## Conclusions

Infants who become overweight are likely to remain overweight as children, but the majority of very heavy infants will have BMI in childhood within the normal range, while most children with BMI above the normal range were not unusually heavy as infants.

## Conflict of interest statement

The authors have no conflicts of interest to disclose.

## Funding Source

Chief Scientist Office, Scotland. TJC is funded by MRC grant MR/M012069/1. LM is based in the Scottish Collaboration for Public Health Research and Policy, which is funded by MRC grant MR/K023209/1. LM is supported by the Farr Institute @ Scotland, which is supported by a 10‐funder consortium: Arthritis Research UK, the British Heart Foundation, Cancer Research UK, the Economic and Social Research Council, the Engineering and Physical Sciences Research Council, the Medical Research Council, the National Institute of Health Research UK, the National Institute for Social Care and Health Research (Welsh Assembly Government), the Chief Scientist Office (Scottish Government Health Directorates), (MRC grant no: MR/K007017/1).

## Financial disclosure statement

The authors have no relevant financial relationships to disclose.

## Supporting information


**Table S1.** weight and BMI status by age, sex and cohort
**Table S2.** Correlation matrix of weight and BMI at different ages. Values are Pearson correlations of Z scores, *boys* upper right, girls lower left
