# Supplementary material for: Pathways into and out of overweight and obesity from infancy to mid‐childhood
Source: Pediatr Obes. 2018 Jul 11;13(10):621–7. doi: 10.1111/ijpo.12427 (PMC6220864; doi:10.1111/ijpo.12427)
Supplement: Supplementary file 1 — Table S1. weight and BMI status by age, sex and cohort Table S2. Correlation matrix of weight and BMI at different ages. Values are Pearson correlations of Z scores, boys upper right, girls lower left [file IJPO-13-621-s001.docx]

Supplementary Table 1: weight and BMI status by age, sex and cohort

|  |  | ***Numbers*** | | | | **% Overweight or obese (≥1 SD)**  **(95% CI)** | | | | **% Obese (≥2 SD)**  **(95% CI)** | | | |
| --- | --- | --- | --- | --- | --- | --- | --- | --- | --- | --- | --- | --- | --- |
|  |  | **GDS** | **GMS** | **Tamp-ere** | **All** | **GDS** | **GMS** | **Tamp-ere** | **All** | **GDS** | **GMS** | **Tamp -ere** | **All** |
| **Weight at** | Boys | 189 | 397 | 623 | 1209 | 31% | 16% | 33% | 27%  (26-28) | 8% | 2% | 7% | 6%  (5.3-6.7) |
| **12 months** | Girls | 203 | 396 | 634 | 1233 | 19% | 20% | 28% | 24%  (23-25) | 4% | 3% | 5% | 4%  (3.4-4.6) |
| **BMI at** | Boys | 182 | 325 | 674 | 1181 | 22% | 35% | 14% | 21%  (20-22) | 6% | 8% | 3% | 5%  (4.4-5.6) |
| **5 years** | Girls | 203 | 353 | 678 | 1234 | 17% | 32% | 16% | 20%  (19-21) | 2% | 9% | 2% | 4%  (3.4-4.6) |
| **BMI at** | Boys | 217 | 275 | 610 | 1102 | 21% | 28% | 24% | 24%  (23-25) | 5% | 9% | 7% | 7%  (6.2-7.8) |
| **8 years** | Girls | 225 | 289 | 601 | 1102 | 17% | 28% | 20% | 21%  (20-22) | 4% | 9% | 4% | 5%  (4.3-5.7) |

Supplementary table 2: Correlation matrix of weight and BMI at different ages

Values are Pearson correlations of Z scores, *boys* upper right, **girls** lower left

|  |  | *boys* |  |  |  |
| --- | --- | --- | --- | --- | --- |
|  |  | Weight  @ 3 m | Weight  @ 12 m | BMI  @ 5 yrs | BMI  @ 8 yrs |
|  | Weight @ 3 m |  | *0.68* | *0.37* | *0.33* |
| **girls** | Weight @ 12 m | **0.70** |  | *0.43* | *0.40* |
|  | BMI @ 5 yrs | **0.44** | **0.55** |  | *0.76* |
|  | BMI @ 8 yrs | **0.38** | **0.49** | **0.79** |  |
